# *METTL14* gene polymorphisms decrease Wilms tumor susceptibility in Chinese children

**DOI:** 10.1186/s12885-021-09019-5

**Published:** 2021-12-04

**Authors:** Zhenjian Zhuo, Rui-Xi Hua, Huizhu Zhang, Huiran Lin, Wen Fu, Jinhong Zhu, Jiwen Cheng, Jiao Zhang, Suhong Li, Haixia Zhou, Huimin Xia, Guochang Liu, Wei Jia, Jing He

**Affiliations:** 1 Department of Pediatric Surgery, Guangzhou Institute of Pediatrics, Guangdong Provincial Key Laboratory of Research in Structural Birth Defect Disease, Guangzhou Women and Children’s Medical Center, Guangzhou Medical University, 9 Jinsui Road, Guangzhou, 510623 Guangdong China; 2 Department of Gynaecology and Obstetrics, Guangzhou Women and Children’s Medical Center, Guangzhou Medical University, Guangzhou, 510623 Guangdong China; 3 Faculty of Medicine, Macau University of Science and Technology, Macau, 999078 China; 4 Department of Clinical Laboratory, Biobank, Harbin Medical University Cancer Hospital, Harbin, 150040 Heilongjiang China; 5 Department of Pediatric Surgery, the Second Affiliated Hospital of Xi’an Jiaotong University, Xi’an, 710004 Shaanxi China; 6 Department of Pediatric Surgery, the First Affiliated Hospital of Zhengzhou University, Zhengzhou, 450052 Henan China; 7 Department of Pathology, Children Hospital and Women Health Center of Shanxi, Shannxi, Taiyuan, 030013 China; 8 Department of Hematology, The Second Affiliated Hospital and Yuying Children’s Hospital of Wenzhou Medical University, Wenzhou, 325027 Zhejiang China

**Keywords:** Wilms tumor, Risk, *METTL14*, Polymorphism, Case-control study

## Abstract

**Background:**

Wilms tumor is a highly heritable malignancy. Aberrant METTL14, a critical component of N6-methyladenosine (m^6^A) methyltransferase, is involved in carcinogenesis. The association between genetic variants in the *METTL14* gene and Wilms tumor susceptibility remains to be fully elucidated. We aimed to assess whether variants within this gene are implicated in Wilms tumor susceptibility.

**Methods:**

A total of 403 patients and 1198 controls were analyzed. *METTL14* genotypes were assessed by TaqMan genotyping assay.

**Result:**

Among the five SNPs analyzed, rs1064034 T > A and rs298982 G > A exhibited a significant association with decreased susceptibility to Wilms tumor. Moreover, the joint analysis revealed that the combination of five protective genotypes exerted significantly more protective effects against Wilms tumor than 0–4 protective genotypes with an OR of 0.69. The stratified analysis further identified the protective effect of rs1064034 T > A, rs298982 G > A, and combined five protective genotypes in specific subgroups. The above significant associations were further validated by haplotype analysis and false-positive report probability analysis. Preliminary mechanism exploration indicated that rs1064034 T > A and rs298982 G > A are correlated with the expression and splicing event of their surrounding genes.

**Conclusions:**

Collectively, our results suggest that *METTL14* gene SNPs may be genetic modifiers for the development of Wilms tumor.

**Supplementary Information:**

The online version contains supplementary material available at 10.1186/s12885-021-09019-5.

## Introduction

Wilms tumor, also known as nephroblastoma, is the most common pediatric kidney cancer [1]. It accounts for over 90% of all the diagnosed kidney tumors in children [2]. The incidence rate of Wilms’ tumor varies geographically [3, 4]. The prevalence of Wilms tumor is about 7 cases per million children in the United States. Wilms tumor is also one of the most common renal tumors in children in China, with an incidence rate of ~ 3.3 per million. Wilms tumors are frequently diagnosed in young children with an average age of 2–3 years [5]. At present, long-term overall survival for the localized Wilms tumors exceeds 90% due to the improved risk stratification-adapted treatment [6]. However, nearly 20% of Wilms tumors are classified into high-risk subtype with frequent metastasis. Patients with high-risk tumors still subject to suboptimal outcomes [7–9]. Chronic health conditions secondary to intensified therapeutic regimens impact nearly 25% of Wilms tumor survivors [10].

The genetics of Wilms tumor tumorigenesis is complex, with multiple oncogenic drivers identified over the years. The currently known repertoire of oncogenic Wilms tumor driver alterations includes mutations in the *WT1*, *CTNNB1*, *TP53*, *AMER1*, as well as an abnormality of 11p15 methylation [11–15]. Apart from these, genetic association analyses in case-control studies also unveiled some Wilms tumor susceptibility loci [16–19]. Nevertheless, the well-established risk factors for Wilms tumor probably are only the tip of the iceberg. So far, all the known gene mutations can only explain less than 50% of Wilms tumor. Therefore, it is imperative to identify more causative variants to improve the understanding of the genetic susceptibility to Wilms tumor. In addition, detailed genetic information leads to new druggable targets, facilitating the development of more effective treatments for Wilms tumor.

N6-methyladenosine (m^6^A) is the most common internal chemical modification on eukaryotic mRNA [20]. m^6^A is mainly involved in the regulation of splicing, subcellular localization, translation, stability, and degradation of mRNA. m^6^A modulators are mainly classified into methyltransferase (writer), demethylase (eraser), and binding protein (reader). Methyltransferases include METTL3, METTL14, and WTAP, which mainly mediate m^6^A methylation of mRNA adenylate. Demethylases, consisting of FTO and ALKBH5, mainly remove m^6^A modification installed on RNA. Binding proteins include YTHDF1/2/3, YTHDC1/2, IGF2BP1/2/3, and eIF3, which are responsible for recognizing bases modified by m^6^A and regulating downstream pathways [21, 22]. The m^6^A modulator proteins play an important role in the occurrence and development of a variety of tumors [23–25]. However, research on the expression and function of m^6^A modulator genes in Wilms tumor has not yet been reported. The scarcity of investigation prompted us to contribute to our current report on associations between genetic variability of *METTL14* and the risk of Wilms tumor. To this end, a total of five common SNPs in the *METTL14* gene were genotyped and tested for their association with Wilms tumor susceptibility.

## Methods

### Sample selection

The study was carried out based on the principles of the Declaration of Helsinki. Approval of the study protocol was obtained from the institutional review board of Guangzhou Women and Children’s Medical Center (Ethics Approval No: 202016600). Eligible cases were all children newly diagnosed with a histologically confirmed Wilms tumor. Controls, recruited from the same hospital, were healthy volunteers of Chinese origin, without family history of Wilms tumor. Written informed consent was signed by all subjects’ guardians. All the subjects were enrolled from March 2001 to March 2018 and were genetically unrelated ethnic Han Chinese from China. A total of 414 cases diagnosed with Wilms tumor and 1199 hospital-based controls were included. They were enrolled from five hospitals (Guangzhou Women and Children’s Medical Center, The Second Affiliated Hospital and Yuying Children’s Hospital of Wenzhou Medical University, The First Affiliated Hospital of Zhengzhou University, Second Affiliated Hospital of Xi’an Jiao Tong University, and Shanxi Provincial Children’s Hospital) in five different cities of China. Detailed information was previously reported [26, 27].

### Polymorphism selection and genotyping

The selection of the five potentially functional *METTL14* gene SNPs (rs1064034 T > A, rs298982 G > A, rs62328061 A > G, rs9884978 G > A, and rs4834698 T > C) was described in detail in our previous studies [28–30]. Genomic DNA from each sample was extracted from peripheral blood. Genotypes were determined using the TaqMan method. Replicate samples (10% of the samples) were picked out of all genotyping batches, and the concordance levels for blind duplicate samples were 100% for all SNPs assayed.

### Statistical analysis

SNP genotypes were tested for consistency with Hardy-Weinberg equilibrium (HWE) within the control sample using a Goodness-of-fit χ^2^ test. Differences between cases and controls in the distribution of demographic and clinical variables were checked using a two-sided χ^2^ test. Adjusted odds ratios (ORs) with 95% confidence intervals (CIs) and two-sided *P*-values were calculated using unconditional logistic regression to estimate the relative risk associated with each genotype. Associations were further estimated in the groups stratified by age, gender, and clinical stages. Haplotype frequency distributions were deduced from observed genotypes using logistic regression analyses [31, 32]. False-positive report probability (FPRP) analysis was applied to assess noteworthy associations with detailed methods presented elsewhere [33, 34]. We performed expression quantitative trait loci (eQTL) and splicing quantitative trait loci (sQTLs) analyses through the Genotype-Tissue Expression (GTEx) project (http://www.gtexportal.org/) to evaluate the correlations between genotypes of candidate SNPs and genes expression as well as alternative splicing (AS) events of genes [35]. A probability value (*P* value) less than 0.05 was considered significant. All statistical analyses were performed using SAS version 9.1 software (SAS Institute, Inc., Cary, North Carolina).

## Results

### Effect of METTL14 gene SNPs on Wilms tumor risk

Clinical characteristics of the participants were depicted in our previous study (Table S1) [27]. Here, we successfully genotyped the five *METTL14* gene SNPs (rs1064034 T > A, rs298982 G > A, rs62328061 A > G, rs9884978 G > A, and rs4834698 T > C) in 403 cases and 1198 controls, out of 414 cases and 1199 controls samples. The correlation between these SNPs and Wilms tumor risk is shown in Table 1. All these SNPs followed Hardy-Weinberg equilibrium (HWE) in controls (HWE *P* > 0.05). The rs1064034 variant alleles were remarkably associated with reduced risk of Wilms tumor (TA vs. TT: adjusted OR = 0.78, 95% CI = 0.61–0.99, *P* = 0.041; TA/AA vs. TT: adjusted OR = 0.83, 95% CI = 0.70–0.995, *P* = 0.044). Similar association was found for the rs298982 (GA/AA vs. GG: adjusted OR = 0.69, 95% CI = 0.53–0.91, *P* = 0.009). We then defined rs1064034 TA/AA, rs298982 GA/AA, rs62328061 AG/AA, rs9884978 GA/GG, and rs4834698 TT/TC as protective genotypes based on their ORs. Participants with 5 protective genotypes showed a 0.69-fold decrease in the risk of developing Wilms tumor when compared with those with 0–4 protective genotypes (95% CI = 0.52–0.91, *P* = 0.008).

**Table 1:** Association between *METTL14* gene polymorphisms and Wilms tumor susceptibility

Genotype	Cases (*N* = 403)	Controls (*N* = 1198)	*P*^a^	Crude OR (95% CI)	*P*	Adjusted OR (95% CI) ^b^	*P*^b^
rs1064034 T > A (HWE = 0.715)							
TT	216 (53.60)	564 (47.08)		1.00		1.00	
TA	152 (37.72)	512 (42.74)		**0.78 (0.61–0.99)**	**0.037**	**0.78 (0.61–0.99)**	**0.041**
AA	35 (8.68)	122 (10.18)		0.75 (0.50–1.13)	0.164	0.76 (0.51–1.15)	0.198
Additive			0.035	**0.83 (0.70–0.99)**	**0.035**	**0.83 (0.70–0.995)**	**0.044**
Dominant	187 (46.40)	634 (52.92)	0.024	**0.77 (0.61–0.97)**	**0.024**	**0.78 (0.62–0.97)**	**0.029**
Recessive	368 (91.32)	1076 (89.82)	0.382	0.84 (0.57–1.24)	0.382	0.86 (0.58–1.27)	0.438
rs298982 G > A (HWE = 0.155)							
GG	321 (79.65)	873 (72.87)		1.00		1.00	
GA	66 (16.38)	292 (24.37)		**0.62 (0.46–0.83)**	**0.001**	**0.62 (0.46–0.84)**	**0.002**
AA	16 (3.97)	33 (2.75)		1.32 (0.72–2.43)	0.375	1.32 (0.72–2.43)	0.373
Additive			0.061	0.80 (0.64–1.01)	0.061	0.81 (0.64–1.02)	0.071
Dominant	82 (20.35)	325 (27.13)	0.007	**0.69 (0.52–0.90)**	**0.007**	**0.69 (0.53–0.91)**	**0.009**
Recessive	387 (96.03)	1165 (97.25)	0.220	1.46 (0.80–2.68)	0.223	1.46 (0.79–2.68)	0.225
rs62328061 A > G (HWE = 0.819)							
AA	281 (69.73)	830 (69.28)		1.00		1.00	
AG	109 (27.05)	333 (27.80)		0.97 (0.75–1.25)	0.796	0.97 (0.75–1.25)	0.812
GG	13 (3.23)	35 (2.92)		1.10 (0.57–2.10)	0.780	1.12 (0.58–2.15)	0.736
Additive			0.963	1.00 (0.81–1.23)	0.963	1.00 (0.81–1.24)	0.998
Dominant	122 (30.27)	368 (30.72)	0.867	0.98 (0.77–1.25)	0.867	0.98 (0.77–1.26)	0.894
Recessive	390 (96.77)	1163 (97.08)	0.757	1.11 (0.58–2.12)	0.757	1.13 (0.59–2.16)	0.714
rs9884978 G > A (HWE = 0.412)							
GG	252 (62.53)	758 (63.27)		1.00		1.00	
GA	131 (32.51)	384 (32.05)		1.03 (0.80–1.31)	0.836	1.03 (0.81–1.31)	0.826
AA	20 (4.96)	56 (4.67)		1.07 (0.63–1.83)	0.791	1.06 (0.62–1.80)	0.826
Additive			0.759	1.03 (0.85–1.25)	0.757	1.03 (0.85–1.25)	0.773
Dominant	151 (37.47)	440 (36.73)	0.790	1.03 (0.82–1.30)	0.789	1.03 (0.82–1.30)	0.791
Recessive	383 (95.04)	1142 (95.33)	0.814	1.07 (0.63–1.80)	0.814	1.05 (0.62–1.78)	0.851
rs4834698 T > C (HWE = 0.827)							
TT	107 (26.55)	329 (27.46)		1.00		1.00	
TC	193 (47.89)	594 (49.58)		1.00 (0.76–1.31)	0.995	0.99 (0.75–1.30)	0.921
CC	103 (25.56)	275 (22.95)		1.15 (0.84–1.58)	0.379	1.14 (0.83–1.56)	0.425
Additive			0.392	1.07 (0.92–1.26)	0.392	1.07 (0.91–1.25)	0.438
Dominant	296 (73.45)	869 (72.54)	0.722	1.05 (0.81–1.35)	0.724	1.03 (0.80–1.34)	0.798
Recessive	300 (74.44)	923 (77.05)	0.287	1.15 (0.89–1.50)	0.287	1.15 (0.88–1.49)	0.304
Combined effect of protective genotypes ^c^							
0–4	322 (79.90)	875 (73.04)		1.00		1.00	
5	81 (20.10)	323 (26.96)	0.006	**0.68 (0.52–0.90)**	**0.006**	**0.69 (0.52–0.91)**	**0.008**

*OR* Odds ratio, *CI* Confidence interval, *HWE* Hardy-Weinberg equilibrium

^a^χ^2^ test for genotype distributions between Wilms tumor patients and controls

^b^Adjusted for age and gender

^c^Protective genotypes were carriers with rs1064034 TA/AA, rs298982 GA/AA, rs62328061 AG/AA, rs9884978 GA/GG and rs4834698 TT/TC

### Stratification analysis of significant SNPs

We analyzed the association between the *METTL14* gene polymorphisms and susceptibility to Wilms tumor in subgroups separated by age, gender, and clinical stages (Table 2). Further stratification study revealed that the rs1064034 was associated with reduced Wilms tumor risk in groups with age > 18 months, female, and clinical stage IV diseases. Moreover, stronger protective effects was found for the GA/AA genotypes of rs298982 and combined five protective genotypes among children age > 18 months, females, clinical stage I + II tumors, and clinical stage III + IV tumors.

**Table 2:** Stratification analysis of protective genotypes with Wilms tumor susceptibility

Variables	rs1064034 (cases/controls)		AOR (95% CI) ^a^	*P*^a^	rs298982 (cases/controls)		AOR (95% CI) ^a^	*P*^a^	Combined (cases/controls)		AOR (95% CI) ^a^	*P*^a^
	TT	TA/AA			GG	GA/AA			0–4	5		
Age, month												
≤ 18	72/243	66/222	1.00 (0.68–1.47)	0.995	105/356	33/109	1.01 (0.65–1.58)	0.971	106/358	32/107	0.99 (0.63–1.56)	0.967
> 18	144/321	121/412	**0.67 (0.50–0.88)**	**0.005**	216/517	49/216	**0.56 (0.39–0.79)**	**0.001**	216/517	49/216	**0.56 (0.39–0.79)**	**0.001**
Gender												
Females	109/251	80/270	**0.68 (0.49–0.95)**	**0.025**	159/394	30/127	**0.59 (0.38–0.91)**	**0.017**	159/396	30/125	**0.60 (0.39–0.93)**	**0.022**
Males	107/313	107/364	0.87 (0.64–1.18)	0.371	162/479	52/198	0.78 (0.55–1.11)	0.172	163/479	51/198	0.76 (0.53–1.09)	0.134
Clinical stages												
I	73/564	64/634	0.81 (0.57–1.15)	0.239	111/873	26/325	0.64 (0.41–1.01)	0.053	111/875	26/323	0.65 (0.42–1.02)	0.060
II	61/564	52/634	0.77 (0.52–1.14)	0.193	88/873	25/325	0.78 (0.49–1.23)	0.285	88/875	25/323	0.79 (0.49–1.25)	0.305
III	44/564	48/634	0.94 (0.61–1.44)	0.781	74/873	18/325	0.64 (0.38–1.10)	0.105	74/875	18/323	0.65 (0.38–1.10)	0.111
IV	28/564	17/634	**0.53 (0.29–0.98)**	**0.043**	37/873	8/325	0.58 (0.27–1.26)	0.171	38/875	7/323	0.50 (0.22–1.13)	0.095
I + II	134/564	116/634	0.79 (0.60–1.04)	0.093	199/873	51/325	**0.70 (0.50–0.98)**	**0.037**	199/875	51/323	**0.71 (0.51–0.99)**	**0.043**
III + IV	72/564	65/634	0.79 (0.55–1.12)	0.183	111/873	26/325	**0.62 (0.40–0.98)**	**0.039**	112/875	25/323	**0.60 (0.38–0.94)**	**0.026**

*AOR* Adjusted odds ratio, *CI* Confidence interval

^a^Adjusted for age and gender, omitting the corresponding factor

### *METTL14* haplotype analysis

We next evaluated whether the haplotypes of the five *METTL14* gene SNPs are linked with Wilms tumor risk (Table 3). When compared to reference haplotype TGAAC, haplotypes AGAGT (*P* = 0.016), AAGGT (*P* = 0.010), and AAAGC (*P* = 0.002) were linked with significantly decreased Wilms tumor risk.

**Table 3:** The frequency of inferred haplotypes of *METTL14* gene based on observed genotypes and their association with the risk of Wilms tumor

Haplotypes ^a^	Cases (*n* = 806)	Controls (*n* = 2396)	Crude OR (95% CI)	*P*	Adjusted OR ^b^ (95% CI)	*P*^b^
TGAAC	78 (9.68)	233 (9.72)	1.00		1.00	
TGAAT	41 (5.09)	111 (4.63)	0.88 (0.57–1.34)	0.542	0.87 (0.57–1.33)	0.516
TGAGC	209 (25.93)	550 (22.95)	0.90 (0.68–1.20)	0.468	0.90 (0.68–1.19)	0.464
TGAGT	242 (30.02)	744 (31.05)	0.77 (0.59–1.02)	0.064	0.77 (0.59–1.02)	0.066
TGGAT	4 (0.50)	0 (0.00)	/	/	/	/
TGGGC	5 (0.62)	1 (0.04)	11.85 (1.37–102.72)	0.025	11.15 (1.28–96.76)	0.029
TGGGT	3 (0.37)	1 (0.04)	7.11 (0.73–69.18)	0.091	7.50 (0.77–73.05)	0.083
TAAAT	1 (0.12)	0 (0.00)	/	/	/	/
TAAGC	1 (0.12)	0 (0.00)	/	/	/	/
AGGAT	23 (2.85)	79 (3.30)	0.69 (0.41–1.16)	0.162	0.70 (0.41–1.16)	0.172
AGGGC	65 (8.06)	193 (8.06)	0.80 (0.55–1.15)	0.227	0.80 (0.55–1.15)	0.221
AGGGT	23 (2.85)	69 (2.88)	0.79 (0.47–1.34)	0.380	0.80 (0.47–1.36)	0.417
AGAAC	3 (0.37)	0 (0.00)	/	/	/	/
AGAAT	2 (0.25)	1 (0.04)	4.74 (0.43–52.87)	0.206	5.23 (0.47–58.94)	0.180
AGAGC	1 (0.12)	1 (0.04)	2.37 (0.15–38.27)	0.543	2.46 (0.15–39.70)	0.527
AGAGT	9 (1.12)	55 (2.30)	**0.39 (0.19–0.82)**	**0.012**	**0.40 (0.19–0.84)**	**0.016**
AAGAC	1 (0.12)	0 (0.00)	/	/	/	/
AAGGC	2 (0.25)	2 (0.08)	2.37 (0.33–17.06)	0.392	2.32 (0.32–16.75)	0.403
AAGGT	9 (1.12)	58 (2.42)	**0.37 (0.18–0.77)**	**0.008**	**0.38 (0.18–0.80)**	**0.010**
AAAAC	0 (0.00)	2 (0.08)	/	/	/	/
AAAAT	18 (2.23)	70 (2.92)	0.61 (0.35–1.08)	0.088	0.62 (0.35–1.09)	0.096
AAAGC	34 (4.22)	162 (6.76)	**0.50 (0.32–0.77)**	**0.002**	**0.50 (0.32–0.77)**	**0.002**
AAAGT	32 (3.97)	64 (2.67)	1.19 (0.73–1.92)	0.492	1.19 (0.73–1.93)	0.488

^a^The haplotypes order were rs1064034, rs298982, rs62328061, rs9884978, and rs4834698

^b^Obtained in logistic regression models with adjustment for age and gender

### False-positive report probability (FPRP) analysis

The obtained significant findings above were further assessed using false-positive report probability (FPRP) analysis (Table 4). At the prior probability of 0.1 and FPRP threshold value of 0.2, the associations between rs1064034 and Wilms tumor risk remained noteworthy in models TA/AA vs. TT and subgroup of children > 18 months in TA/AA vs. TT. Noteworthy results were also found for the GA vs. GG, GA/AA vs. GG, and subgroup of children > 18 months in GA/AA vs. GG. In addition, a significant decrease of Wilms tumor risk was detected in the carrier of 5 vs. 0–4 protective genotypes and subgroup of children > 18 months in 5 vs. 0–4 protective genotypes. Significant findings remained noteworthy in the haplotype TGGGC when compared to reference haplotype TGAAC.

**Table 4:** False-positive report probability analysis for significant findings

Genotype	OR (95% CI)	*P*^a^	Statistical power ^b^	Prior probability				
				0.25	0.1	0.01	0.001	0.0001
rs1064034 T > A								
TA vs. TT	0.78 (0.61–0.99)	0.0372	0.899	**0.110**	0.271	0.804	0.976	0.998
TA/AA vs. TT	0.77 (0.61–0.97)	0.0237	0.886	**0.074**	**0.194**	0.726	0.964	0.996
> 18	0.66 (0.49–0.87)	0.0033	0.441	**0.022**	**0.063**	0.426	0.882	0.987
Females	0.68 (0.49–0.96)	0.0257	0.544	**0.124**	0.298	0.824	0.979	0.998
Stage IV	0.54 (0.29–0.997)	0.049	0.255	0.366	0.634	0.950	0.995	0.999
rs298982 G > A								
GA vs. GG	0.62 (0.46–0.83)	0.0013	0.307	**0.013**	**0.037**	0.295	0.809	0.977
GA/AA vs. GG	0.69 (0.52–0.90)	0.0071	0.571	**0.036**	**0.101**	0.552	0.926	0.992
> 18	0.54 (0.38–0.77)	0.0006	0.134	**0.013**	**0.039**	0.308	0.818	0.978
Female	0.59 (0.38–0.91)	0.0167	0.287	**0.149**	0.344	0.852	0.983	0.998
Stage I	0.63 (0.40–0.98)	0.0416	0.399	0.238	0.484	0.912	0.990	0.999
Stage I + II	0.69 (0.49–0.96)	0.028	0.566	**0.129**	0.308	0.830	0.980	0.998
Stage III + IV	0.63 (0.40–0.98)	0.0416	0.400	0.238	0.484	0.911	0.990	0.999
Protective genotypes								
5 vs. 0–4	0.68 (0.52–0.90)	0.0063	0.552	**0.033**	**0.093**	0.531	0.919	0.991
> 18	0.54 (0.38–0.77)	0.0006	0.134	**0.013**	**0.039**	0.308	0.818	0.978
Female	0.60 (0.39–0.93)	0.0216	0.318	**0.169**	0.379	0.871	0.985	0.999
Stage I	0.64 (0.41–0.99)	0.0455	0.413	0.248	0.498	0.916	0.991	0.999
Stage I + II	0.69 (0.50–0.97)	0.0318	0.585	**0.140**	0.329	0.843	0.982	0.998
Stage III + IV	0.61 (0.39–0.95)	0.0291	0.338	0.205	0.437	0.895	0.989	0.999
Haplotypes								
TGGGC vs. TGAAC	11.85 (1.37–102.72)	0.025	0.035	0.683	0.866	0.986	0.999	1.000
AGAGT vs. TGAAC	0.39 (0.19–0.82)	0.012	0.089	0.295	0.557	0.932	0.993	0.999
TGGGC vs. TGAAC	0.37 (0.18–0.77)	0.008	0.070	0.256	0.508	0.919	0.991	0.999
TGGGC vs. TGAAC	0.50 (0.32–0.77)	0.002	0.148	**0.035**	**0.099**	0.547	0.924	0.992

*OR* Odds ratio, *CI* Confidence interval

^a^Chi-square test was used to calculate the genotype frequency distributions

^b^Statistical power was calculated using the number of observations in each subgroup and the corresponding ORs and *P* values in this table

### Effect of SNPs on gene expression (eQTLs) and splicing (sQTLs)

**Fig. 1:**
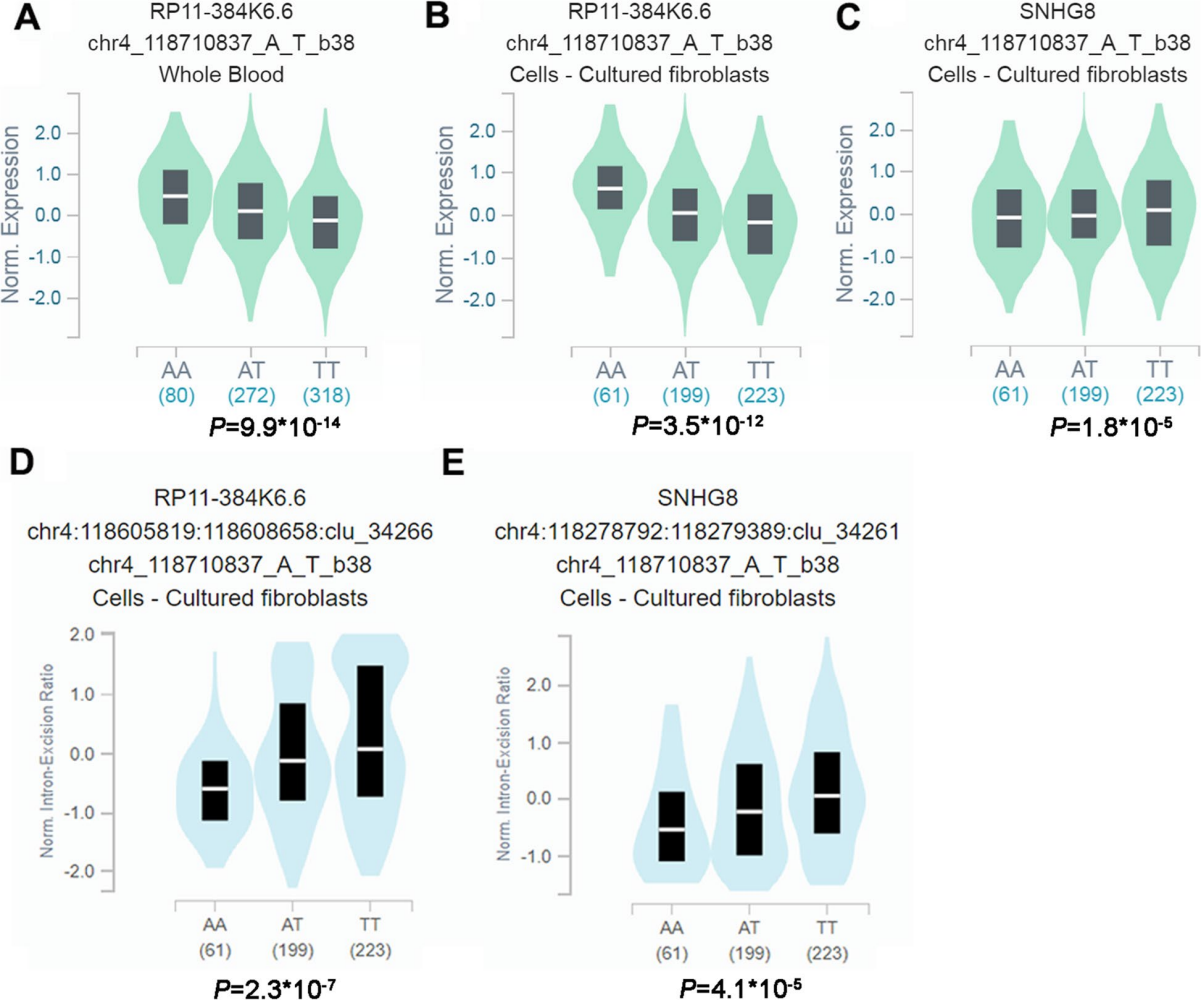
Functional relevance of rs1064034 on gene expression and splicing events in GTEx database. rs1064034 was significantly associated with *RP11-384 K6.6* level in the **A** whole blood (*P* = 9.9*10^−14^) and **B** cells-cultured fibroblasts (*P* = 3.5*10^−12^) as well as **C***SNHG8* mRNA level in the cells-cultured fibroblasts (*P* = 1.8*10^−5^). rs1064034 can affect the splicing events of **D***RP11-384 K6.6* (*P* = 2.3*10^−7^) and **E***SNHG8* (*P* = 4.1*10^−5^) genes in cells-cultured fibroblasts

**Fig. 2:**
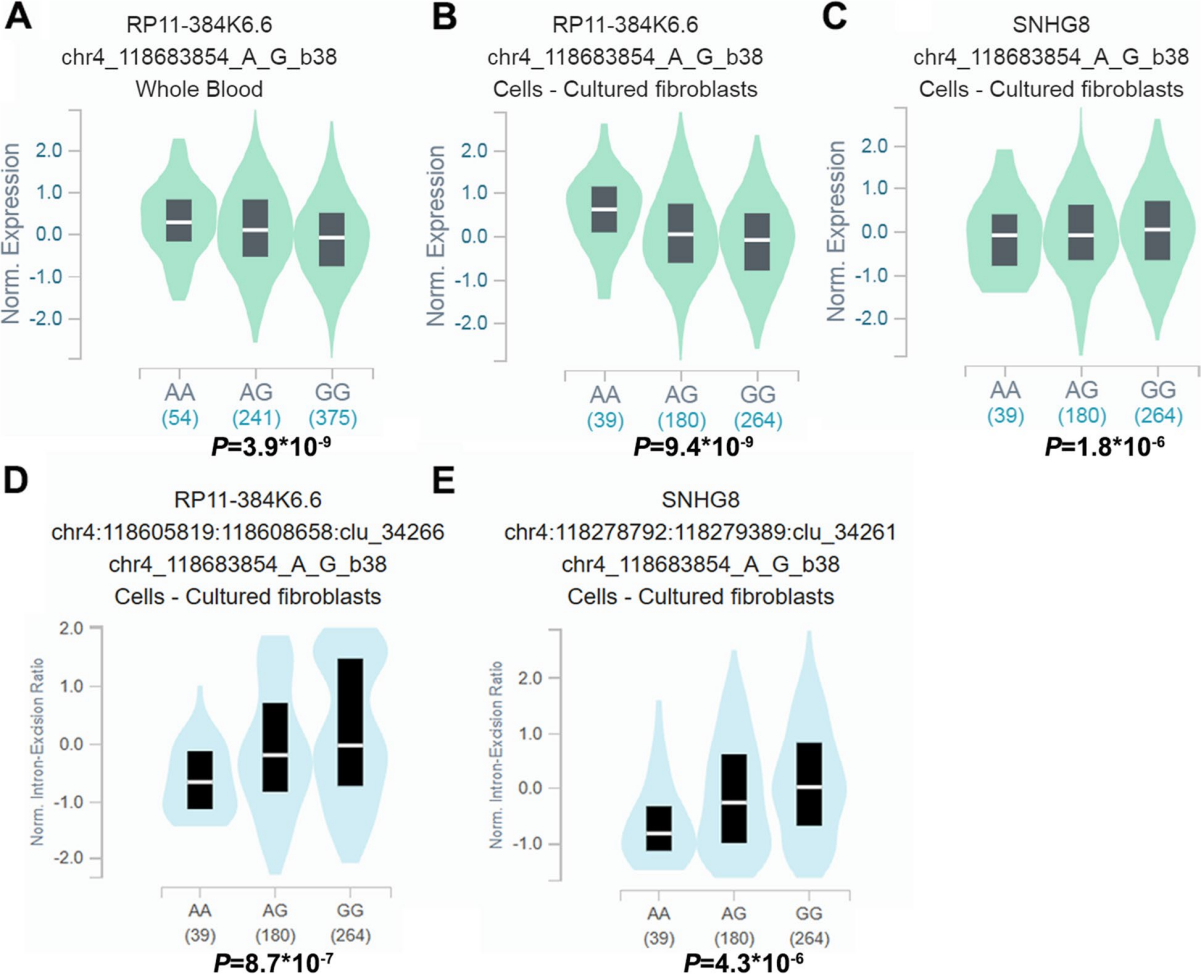
Functional relevance of rs298982 on gene expression and splicing events in GTEx database. rs298982 was significantly associated with *RP11-384 K6.6* level in the **A** whole blood (*P* = 3.9*10^−9^) and **B** cells-cultured fibroblasts (*P* = 9.4*10^−9^) as well as **C***SNHG8* mRNA level in the cells-cultured fibroblasts (*P* = 1.8*10^−6^). rs1064034 can affect the splicing events of **D***RP11-384 K6.6* (*P* = 8.7*10^− 7^) and **E***SNHG8* (*P* = 4.3*10^− 6^) genes in cells-cultured fibroblasts

We further used GTEx to analyze the expression quantitative trait loci (eQTLs) and splicing quantitative trait loci (sQTLs) of rs1064034 and rs298982. Interestingly, rs1064034 was significantly associated with mRNA expression of *RP11-384 K6.6* in the whole blood (Fig. 1A) and cells-cultured fibroblasts (Fig. 1B), as well as *SNHG8* in cells-cultured fibroblasts (Fig. 1C). We found that the rs1064034 could affect the splicing events of *RP11-384 K6.6* (Fig. 1D) and *SNHG8* (Fig. 1E) genes in cells-cultured fibroblasts. Similarly, rs298982 was significantly associated with mRNA expression of *RP11-384 K6.6* in the whole blood (Fig. 2A) and cells-cultured fibroblasts (Fig. 2B), as well as *SNHG8* in cells-cultured fibroblasts (Fig. 2C). SNP rs298982 could also affect the splicing events of *RP11-384 K6.6* (Fig. 2D) and *SNHG8* (Fig. 2E) genes in cells-cultured fibroblasts.

## Discussion

This is the first genetic epidemiological study on the association of genetic variants in the *METTL14* gene and Wilms tumor risk. We found that common variants in the *METTL14* gene were significantly associated with susceptibility to this malignancy. This study may contribute to uncovering the underlying biology and genetics of Wilms tumor.

METTL14 is a key component of the m^6^A methyltransferase complex. METTL14 has different roles in different tumors and can be either a cancer promoter or suppressor. Chen et al. [36] identified METTL14 as a tumor suppressor in colorectal cancer. The low METTL14 was significantly associated with poor overall survival. Further functional experiments demonstrated that METTL14 inhibited the progression of colorectal cancer by regulating the production process of m^6^A-dependent precursor miR-375. Ma et al. [37] found that METTL14 was remarkedly downregulated in hepatocellular carcinoma. The reduced METTL14 expression was significantly associated with unfavorable recurrence-free survival and overall survival. The inhibitory role of METTL14 on hepatocellular carcinoma may be partly attributed to its facilitation of the primary miR-126 maturation in a m^6^A-dependent manner. METTL14 exerted an oncogenic role in acute myeloid leukemia via mRNA m^6^A modification [38]. Lang et al. [39] observed that METTL14 was an important driver in EBV-induced oncogenesis. They found that knockdown of METTL14 caused a decreased tumorigenic activity of EBV-transformed cells in the xenograft animal model systems. METTL14 could promote the growth and metastasis of pancreatic cancer by up regulating the m^6^A level of PERP mRNA [40].

Since the function and mechanism of m^6^A modification in mammals have not been studied for a long time, the effect of SNPs of m^6^A modification genes on genetic susceptibility to tumors has been hardly understood. Through adopting a two-stage case-control study, Meng et al. [41] conducted the first study to explore whether m^6^A gene SNPs could predispose to colorectal cancer in the Chinese population. All the five *METTL14* gene SNPs (rs115267066, rs167246, rs2029399, rs298981, and rs441216) failed to show impacts on colorectal cancer risk. By enrolling 898 patients with neuroblastoma and 1734 controls, our group found that the *METTL14* gene rs298982 G > A and rs62328061 A > G could significantly reduce the risk of neuroblastoma in children, while rs9884978 G > A and rs4834698 T > C could significantly increase the risk of neuroblastoma [28]. Regarding Wilms tumor, no studies investigating the role of *METTL14* gene SNPs were available by far.

**Fig. 3:**
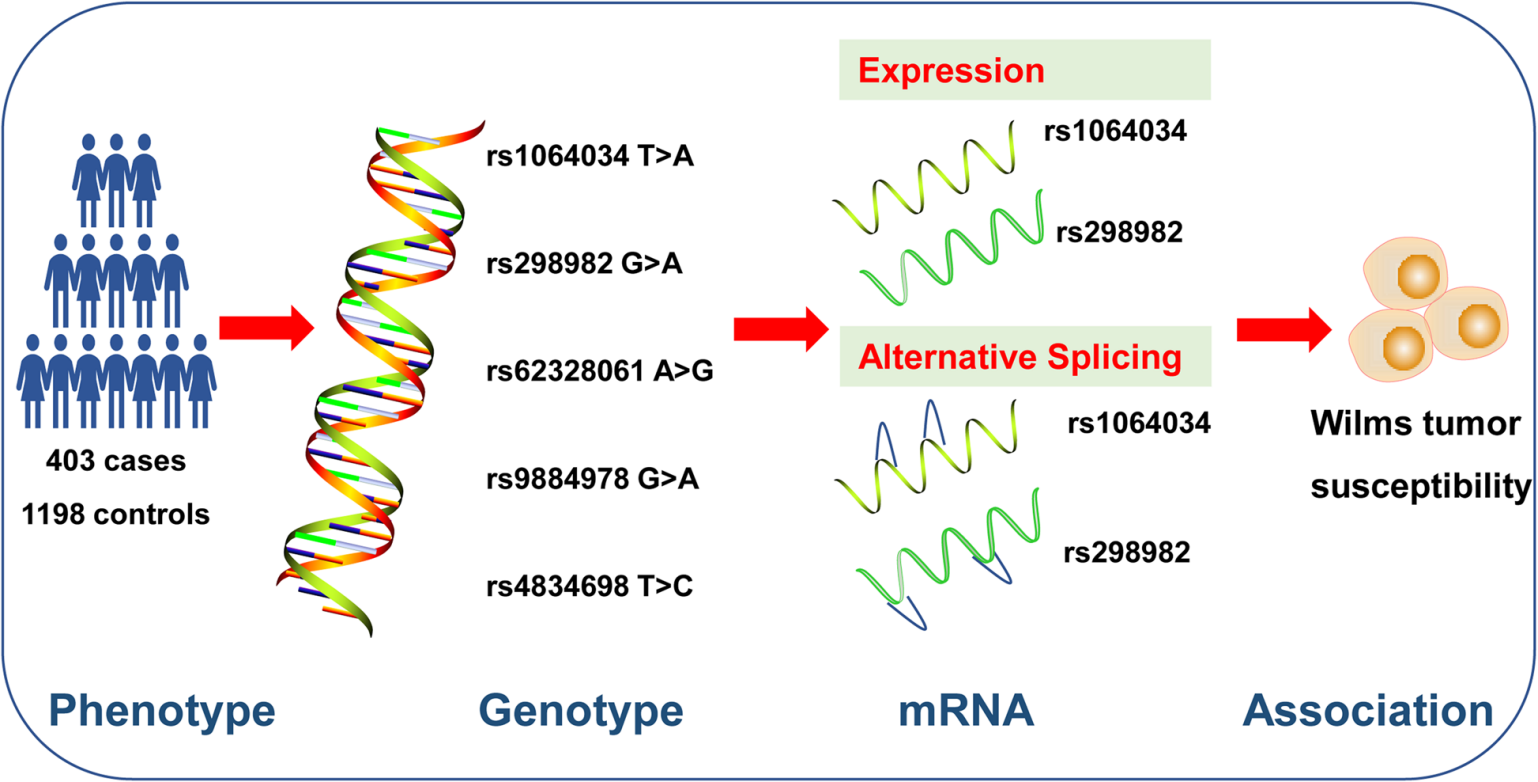
Possible mechanism of how SNPs rs1064034 and rs298982 confer to Wilms tumor risk

In the current study, rs1064034 and rs298982 variant alleles were found to protect from developing Wilms tumor. The combination of five protective genotypes led to a 0.69-fold decrease in the risk of developing Wilms tumor in comparison to 0–4 protective genotypes, indicating the stronger effect of the combined SNPs. It is believed that association studies based on haplotypes of multiple SNPs instead of individual SNP remarkedly strengthen the power for mapping and characterizing disease-causing genes [42, 43]. Thus, we examined whether haplotypes of *METTL14* gene are associated with Wilms tumor risk. Expectedly, *METTL14* gene haplotypes showed a significantly increased protection against Wilms tumor, indicating the synergistic effects of these SNPs. Genetic variation can modulate gene expression, thereby affecting phenotypes and susceptibility to complex diseases such as Wilms tumor. Here we harnessed the GTEx database to evaluate the effect of SNPs rs1064034 and rs298982 on expression and alternative splicing events of genes. We found that rs1064034 and rs298982 were significantly correlated with the expression and splicing of its nearby genes *SNHG8* and *RP11-384 K6.6*. LncRNA *SNHG8* acts as a vital role in tumorigenesis [44–48]. Thus, it is biologically possible that changes of the expression and splicing of *SNHG8* and *RP11-384 K6.6* caused by SNP rs1064034 and rs298982 may influence Wilms tumor risk (Fig. 3). Our results bring new insights into genetic mechanisms of how *METTL14* affects Wilms tumor risk. Our findings identify *METTL14* gene SNPs as risk markers in pediatric Wilms tumor. These findings not only show the relationship between some *METTL14* gene SNPs and Wilms tumor risk but also can help to improve risk stratification strategies for Wilms tumor patients. In all, in-depth mechanism of how *METTL14* SNPs affects Wilms tumor risk by regulating the gene expression and splicing pattern awaits to be elucidated. Potential limitations of our study include relatively small sample size, a lack of independent validation, and failure to incorporate other confounders. We also acknowledged that the conclusion obtained here was limited to Chinese. Cautions should be taken when interpreting this conclusion in other populations.

## Conclusion

In summary, we demonstrated the significant effects of *METTL14* gene SNPs on the risk of Wilms tumor. However, further validation studies with larger sample size and involving different populations are required to strengthen this association.

## Supplementary Information

**Additional file 1: Table S1*.*** Frequency distribution of selected variables in Wilms tumor patients and cancer-free controls.

## Data Availability

All data and material are available from the corresponding author on reasonable request. The datasets generated or analyzed during the current study are not publicly available but are available with the corresponding author and can be provided on reasonable request.

## Abbreviations

m^6^A: N6-methyladenosine
HWE: Hardy-Weinberg equilibrium
ORs: Odds ratios
CIs: Confidence intervals
FPRP: False-positive report probability analysis
eQTL: Expression quantitative trait loci
sQTLs: Splicing quantitative trait loci
GTEx: Genotype-Tissue Expression
AS: Alternative splicing
